# Editorial: Reconfiguration of neuronal ensembles throughout learning

**DOI:** 10.3389/fnsys.2023.1161967

**Published:** 2023-03-14

**Authors:** Luis Carrillo-Reid, Masakazu Agetsuma, Emilio Kropff

**Affiliations:** ^1^Neurobiology Institute, National Autonomous University of Mexico, Juriquilla, Queretaro, Mexico; ^2^Institute for Quantum Life Science, Quantum Regenerative and Biomedical Engineering Team, Chiba, Japan; ^3^Leloir Institute-IIBBA/CONICET, Buenos Aires, Argentina

**Keywords:** neuronal ensemble, calcium imaging, electrical recordings, attractor network model, pathological states

Learning is a continuous process that updates new information to previous experiences (Eichenbaum, 2000). It has been proposed that learning processes involve changes in the brain that generate reliable patterns of activity (Frankland et al., 2019). The development of recording techniques that allow the identification of neurons participating in learning processes, suggests that memories are stored by interactive groups of neurons (Han et al., 2007). In this context, a neuronal ensemble could be defined as a group of neurons with coordinated activity whose activation drives a learned behavior (Carrillo-Reid and Yuste, 2020). Reliable patterns of activity related to memories emerge by the reconfiguration of neuronal ensembles mediated by the intrinsic properties of neurons, the functional connectivity of ensemble members, and the canonical circuits composing ensembles that are constantly changing throughout time (Carrillo-Reid, 2022). It has been shown that neuronal ensembles are altered in pathological states (Jaidar et al., 2010; Hamm et al., 2017) possibly reflecting long term adaptations to compensate the loss of function caused by disease. Thus, in healthy conditions recurrent activity patterns become functional memories, whereas in pathological conditions recurrent activity patterns give rise to behavioral impairments.

The articles in this Research Topic explore how the neuronal ensemble framework could be applied to understand changes in brain circuits, using simultaneous electrical recordings of different brain areas, attractor models of memory function, and dimensionality reduction techniques of calcium imaging population activity. This Research Topic consists of two original research articles, one methods article, and a mini review that in conjunction accentuate the importance of using different computational and experimental approaches to understand population activity in health and disease, as summarized below.

The recent development of optical and analytical approaches to study neuronal microcircuits with single cell resolution has allowed the characterization of neuronal ensemble activity in different experimental conditions (Carrillo-Reid et al., 2017). It has been shown using calcium imaging that the coordinated activity of neuronal ensembles could be related to sensory stimuli, perception, movement, memory, and learning processes (Carrillo-Reid et al., 2019; Jaidar et al., 2019; Robinson et al., 2020). However, the application of such approaches for clinical purposes is still under development. Lara-González et al. present a comprehensible mini review summarizing a conceptual framework for the study of parkinsonian neuronal ensembles from synaptic properties to functional modules in brain slices. Such mini review emphasizes that the circuit properties described on *ex-vivo* brain tissue could be used as biomarkers for testing drugs with therapeutical potential.

Similarly, calcium imaging population recordings with single cell resolution could be understood as multidimensional data arrays that contain information about sequential activity patterns of neuronal ensembles (Carrillo-Reid and Yuste, 2020). To exploit the information contained in such recordings, Serrano-Reyes et al. provide a methods article proposing a pipeline to study neuronal population activity from calcium imaging recordings in pathological brain microcircuits. Implementing different algorithms to identify and describe the properties of neuronal ensembles they conclude that such pipeline could be used to analyze brain biopsies from patients as a pre-clinical bioassay. Such bioassays could then be used to test the effect of different pharmacological manipulations aiming to revert pathological activity patterns by understanding the microcircuit properties of individualized brain tissue.

On the other hand, population electrical recordings could be used to measure unique features that result from the synchronous activity of neuronal ensembles (Buzsaki, 2010). It has been shown that sharp-wave ripples represent the coordinated activity of neurons (Wilson and McNaughton, 1994), and that the disruption of sharp wave ripples are related to the impairment of memory processes in Alzheimer's disease (Jones et al., 2019). Funane et al. present an original research article reporting the loss of coordination of sharp-wave ripples between the medial entorhinal cortex and hippocampal CA1 in a mouse model of Alzheimer's disease. The fact that such loss of coordination could be observed before memory impairments highlights that electrical activity could be used as a biomarker for the early diagnosis of the disease.

The understanding of how memories are stored began with the research of Semon and Lashley proposing that engrams could represent the place where memories reside (Josselyn et al., 2015). The refinement of optical and molecular techniques to label and follow the activity of neurons suggests that the interaction of neuronal ensembles underlie memories (Ghandour et al., 2019). However, the relationship between wiring and memory is hard to study experimentally. Emina and Kropff contribute an original research article implementing a computational model of an autoassociative attractor network. Through simulations, they show that selective re-wiring taking place in parallel to the learning process can produce an enhancement in the storage capacity of the network of up to one order of magnitude, overcoming the limitations in storage capacity observed in randomly connected networks.

Overall, these articles emphasize that an integrated framework for the study of neuronal ensembles requires the combination of computational models, population analyses, and the description of the interaction between different brain nuclei (Carrillo-Reid and Calderon, 2022). The further development of recoding and analytical techniques to characterize the properties of neuronal ensembles throughout time will allow a deeper understanding of the brain reconfigurations that underly learning processes.

## Author contributions

LC-R wrote the original draft. LC-R, MA, and EK edited the final version of the manuscript. All authors contributed to the article and approved the submitted version.
